# Effects of Regulatory Focus and Emotions on Information Preferences: The Affect-as-Information Perspective

**DOI:** 10.3389/fpsyg.2020.01397

**Published:** 2020-07-10

**Authors:** Xiaomei Wang, Quanquan Zheng, Jia Wang, Yangli Gu, Jiongying Li

**Affiliations:** ^1^School of Media Studies and Humanities, Zhejiang University City College, Hangzhou, China; ^2^Department of Psychology and Behavioral Sciences, Zhejiang University, Hangzhou, China

**Keywords:** regulatory focus, emotion, affect-as-information, information preference, Implicit Association Test

## Abstract

This study explored the effects of regulatory focus and emotions on information preferences, specifically information selection preferences (experiment 1) and implicit information preferences (experiment 2). Our findings revealed that, in the promotion-focused condition, individuals preferred hedonic information (vs. functional information) when they were happy (vs. sad). However, emotions’ effects on information preferences were attenuated in the prevention-focused condition. In experiment 3, we tested whether regulatory focus and salient emotions influenced information preferences. The results suggested that regulatory focus and salient emotions had no significant interactive effect on information selection preferences, but had a significant interactive effect on implicit preferences. These results further our understanding of the psychological dynamic mechanism involved in information preferences, which augments the affect-as-information theory.

## Introduction

With the development of network technology in the information age, it has become important to identify what factors affect people’s information preferences. Studies have shown that regulatory focus affects information searches, information sharing, and information utilization in the decision-making process (Higgins, 1999; Ciuchta et al., 2016; Ewe et al., 2018). People systematically prefer information consistent with their beliefs, attitudes, and decisions. Higgins (2006) found that emotions also have a regulatory focus function, another key factor involved in the process of constructing individual values and attitudes. When people evaluate and judge things, they often regard their current emotion as an information resource and ask themselves, “How do I feel about this?” People decide how much they like things based on their own emotional experiences. As information, antecedent residual emotional states often imbue the evaluation of irrelevant things. Schwarz and Bohner (1996) proposed the affect-as-information model, stating that emotions can provide information related to one’s current available tendencies and cognitions; people use emotional information to make “colored” judgments that influence their subsequent attitudes and behaviors. In a classic study by Schwarz and Clore (1983), weather-induced emotions were used as a factor in assessing people’s life satisfaction, and the results indicated that people evaluated their satisfaction higher on sunny days than on rainy days. However, when the participants were asked about the weather at the beginning of the experiment, they tended to attribute their emotions to the weather; subsequently, their emotions were not used as information to rate their life satisfaction. When people feel that emotions are related to evaluations and judgments, they then rely on emotions to make judgments (Pham, 1998). When the emotion’s source is made salient, its influence on judgments and decision-making tends to decrease or disappear (Raghunathan et al., 2006; Hasford et al., 2015). In this study, we explored the presence of emotions both with and without salient sources to identify how they can influence an individual’s information preferences in the promotion and prevention focus conditions.

Research has shown that people may have two different attitudes toward an object at the same time: explicit and implicit (Greenwald and Bannji, 1995). Explicit attitudes can be directly assessed through self-reported questionnaires or interviews, but due to the limitations of consciousness or motivation, the self-report approach cannot capture all attitudes well (Jones et al., 2002). Implicit attitudes are described as evaluative responses to an object, which are measured indirectly; that is, individuals may not be aware of their implicit attitudes, may be unable to verbalize them, or may be unwilling to express their thoughts. The Implicit Association Test (IAT), however, can automatically measure relative attitudinal preferences between two categories, which makes the test scores resistant to deception (Greenwald et al., 2009). Notably, in previous studies, the influence of regulatory focus on preference was discussed at the level of explicit preference (Voss et al., 2003; Pham and Avnet, 2004; Roy and Ng, 2012; Mantovani et al., 2018). To fill this gap, we used the IAT to probe the implicit information preferences of promotion- and prevention-focused individuals in the presence of emotions with or without salient sources.

## Theoretical Background and Hypotheses

### Regulatory Focus and Emotions

Higgins (1997) argued that people have a tendency to move toward certain goals with one of two different regulatory focuses: promotion or prevention. Different regulatory focus types imply different sensitivities to information about an emotion (Shah and Higgins, 2001). Promotion-focused individuals pursue growth-related outcomes and are more likely to experience happiness, while prevention-focused individuals pursue safety-related outcomes and are more likely to experience sorrow (Higgins, 1998; Ouyang et al., 2015; Song and Qu, 2019). Previous studies found that promotion focus can induce heuristic processing, which enables people to make judgments based on subjective emotions, while prevention focus can induce analytic processing, which promotes judgments less based on subjective emotions (Friedman and Förster, 2000, 2001; Pham et al., 2001). Pham and Avnet (2004) suggested that promotion-focused individuals place more weight on their subjective emotional responses during product evaluation processes, while prevention-focused individuals place more weight on rational information. Baek and Reid (2013) claimed that emotions and regulatory focus have a significant interaction effect on individuals’ attitudes and willingness to sponsor behavior. When information is framed with a promotion focus, a happy mood fosters a more positive attitude and greater willingness than does a sad mood. In contrast, when information is framed with a prevention focus, the effects of emotions on attitude and willingness are attenuated. Thus, we proposed the following hypotheses:

Hypothesis 1a (H1a): In the promotion focus condition, an individual’s different emotions will generate different information preferences.

Hypothesis 1b (H1b): In the prevention focus condition, emotions will affect information preferences less than in the promotion focus.

### Regulatory Focus and Salience of Emotions

The affect-as-information theory states that a particular emotion has informational value for a current goal or task. People often assume that, unless they are aware of the emotion’s source, emotions are related to what they are focusing on (Higgins, 1998). That is, an emotion’s influence depends on the salience of the emotion’s source. Gasper (2004) suggested that affect-as-information may influence perceptual focus; for example, when emotions seem irrelevant to the task, sadness promotes a more local or less global focus than does happiness. However, the emotion’s effect on perceptual focus is eliminated when emotions seem relevant to the task. Isbell and Lair (2013) found that, without a salient emotional source, individuals tend to use abstract concepts when they are happy. For example, happy people may respond with “I am a human being” or “I am honest” when asked “Who are you?,” whereas people tend to use concrete terms when they are sad (e.g., “I am hungry” or “I am sitting at a desk”). However, when the emotion’s source is made salient, people’s emotions no longer influence their representations of the self. Thus, we predicted the following:

Hypothesis 2 (H2): When an emotion’s source is made salient, the interaction between emotions and regulatory focus on information preference will be mitigated.

## Experiment 1

To test hypothesis 1, we conducted experiment 1 to examine the influences of regulatory focus and emotions on information selection preferences.

### Participants and Design

Sixty-six undergraduate and graduate students (42 women and 24 men) agreed to participate in this experiment. The participants were randomly assigned to a 2 (regulatory focus: promotion focus or prevention focus) × 2 (emotion: happy or sad) between-subjects design. On completing the experiment, all the participants received a gift worth CNY 15 yuan.

### Materials and Procedure

The participants first completed a regulatory focus manipulation task after being randomly assigned to either the promotion-focused or the prevention-focused group. During this process, the participants spent 5–10 min writing a paragraph describing their personal criteria (thoughts or ideals). In the promotion focus condition, the participants were asked to “describe how your hopes and aspirations are different now from when you were growing up.” Those in the prevention condition were asked to “describe how your duties and obligations are different now from when you were growing up” (Freitas and Higgins, 2002).

After the regulatory focus manipulation was complete, the participants completed an emotion-priming task by recalling a happy or a sad event and writing down as many details as possible within 5 min. Next, to enhance the effect of activating emotions, the participants were instructed to view a waist-up photo of a man in a plain white T-shirt with no jewelry in front of a gray background expressing happiness or sadness (Tracy et al., 2009). The priming instructions read, “Look carefully at the picture. What emotions do you think the person in the picture is expressing? Please come up with 3–5 words that best describe the emotion expressed by the person.” Because previous studies have shown that these emotion-priming materials were very effective (Tracy et al., 2009; Griskevicius et al., 2010), and given that we did not want to increase the salience of the emotion in the induction process, the experiments did not include an emotional manipulation check.

Following the emotion induction task, the participants completed the information selection task. In order to prepare this formal experimental material, a preliminary experiment was conducted. A total of 26 university students participated in a survey in which the participants categorized functional and hedonic information; functional information refers to instrumental and practical types of information, whereas hedonic information encompasses aesthetic and experiential types of information (Chitturi et al., 2007). The pilot study revealed the top four items singled out: functional information included economic news, political news, lifestyle tips, and weather forecasts, and hedonic information included entertainment information, art appreciation, movie information, and music messages. Therefore, in the formal experiment, there were four different selection scenarios, each of which presented two possible options. The scenarios were elicited with the statement: “…here are four different options. If you had 10 mins, which one would you choose to watch? Please choose between A and B according to your current thoughts: A. economic information; B. entertainment information.” The participants were asked to choose one option to represent their information selection preference. For example, if the participant chose A, it was denoted as 1 point on the functional information preference index.

### Results

The results are displayed in Table 1. As expected, there was a significant interaction effect between emotions and regulatory focus on information selection preference [*M*_happy_ = 1.53, *SD* = 0.51; *M*_sad_ = 1.78, *SD* = 0.42; *M*_prom_ = 1.69, *SD* = 0.47; *M*_prev_ = 1.61, *SD* = 0.49; *F*_(1,62)_ = 4.67, *p* < 0.05], and the main effect of emotions was also significant [*F*_(1,62)_ = 5.22, *p* < 0.05]. We conducted a simple effect analysis to further understand the effect of the interaction between emotions and regulatory focus on information selection preference. The results indicated that, in the promotion-focused condition, there was a more significant difference for the influence of a happy emotion on information selection preference [*M*_happy_ = 1.44, *SD* = 0.51; *M*_sad_ = 1.94, *SD* = 0.25; *F*_(1,62)_ = 9.61, *p* < 0.01] compared to a sad emotion. The simple effect analysis revealed that, in the promotion-focused condition, individuals preferred hedonic information when they are happy, but functional information when they are sad. These results supported our first hypothesis. However, in the prevention-focused condition, there was no significant difference for the influence of emotions on information selection preference [*M*_happy_ = 1.61, *SD* = 0.50; *M*_sad_ = 1.63, *SD* = 0.50; *F*_(1,62)_ = 0.01, *p* = 0.93].

**TABLE 1:** Analysis of variance of the effect of emotions and regulatory focus on information selection preference.

Variables	*df*	MS	*F*	*p*	Partial *η*^2^
Emotion	1	1.09	5.22	0.03	0.08
Regulatory focus	1	0.08	0.38	0.54	0.01
Emotion × Regulatory focus	1	0.97	4.67	0.04	0.07
Error	62	0.21			

### Discussion

The results obtained in experiment 1 demonstrated that people’s emotions interacted with their regulatory focus in information selection preference. In the promotion-focused condition, people were more dependent on their emotions for information selection purposes. However, in the prevention-focused condition, people’s information selections were not easily affected by their emotions. These results are consistent with prior research; for instance, Pham and Avnet (2009) suggested that promotion-focused individuals weigh affective inputs more heavily in their judgments and decision-making processes in comparison to prevention-focused individuals. The promotion focus, which is characterized by eagerness, should encourage one’s reliance on emotions in heuristic processing. Alternatively, the prevention focus, which is characterized by vigilance, should reduce one’s reliance on emotions in analytical processing (Friedman and Förster, 2000, 2001).

Experiment 1 investigated how regulatory focus and emotions influenced information selection preferences, assessed *via* self-report measures. Experiment 2 further investigated the effects of regulatory focus and emotions on implicit information preferences, measured by the IAT.

## Experiment 2

We conducted experiment 2 using the IAT to examine the influences of regulatory focus and emotions on implicit information preferences.

### Participants and Design

The participants consisted of 63 undergraduate and graduate students (38 women and 25 men), who were provided with small gifts worth CNY 15 yuan for their participation. Data from 58 participants were analysed, 5 invalid responses were deleted due to data discrepancies (such as the same option for all questions). The participants were randomly assigned to a 2 (regulatory focus: promotion or prevention focus) × 2 (emotion: happy or sad) between-subjects design.

### Procedure

The participants first completed a regulatory focus manipulation task. Previous research has shown that role-playing scenarios activate regulatory focus (Jain et al., 2007; Chung and Han, 2013). We manipulated regulatory focus by requiring participants to unscramble six jumbled words that are names of commonly used cosmetic brands. Promotion condition participants were told: “You will win two points for each correct name. You will not gain points if you do not get a correct name. Your goal is to win as many points as possible by maximizing the number of names you get correct.” Prevention condition participants were told: “You will lose two points for each wrong name. You will not lose points if you get a correct name. Your goal is to lose as few points as possible by maximizing the number of names you get correct.” After finishing the regulatory focus manipulation task, the participants completed the same priming emotion task that was conducted in the first experiment. Then, the participants completed implicit information preference measurements through the IAT on computers.

In the IAT, hedonic and functional information were used as target categories, and the attribute words comprised positive and negative adjectives. During the test, the computer program automatically recorded the participants’ correct rates and reaction times. *D* scores were used to calculate the IAT effect (Greenwald et al., 2003). A high *D* value means more hedonic preferences. As illustrated in Table 2, the IAT was made up of seven blocks of trials.

**TABLE 2:** Sequence of blocks for the IAT experiment.

Blocks	Categories for “E” key	Categories for “I” key	Number of trials
(1) Single categorization of target word (practice)	Hedonic information	Functional information	20
(2) Single categorization of target word (practice)	Positive	Negative	20
(3) Combined categorization (practice)	Positive/hedonic information	Negative/functional information	20
(4) Combined categorization (test)	Positive/hedonic information	Negative/functional information	40
(5) Single categorization of target word (reversed)	Functional information	Hedonistic information	20
(6) Combined categorization (practice, reversed)	Positive/functional information	Negative/hedonic information	20
(7) Combined categorization (test, reversed)	Positive/function information	Negative/hedonic information	40

### Results

The results displayed in Table 3 indicated that there was a significant interaction effect between emotions and regulatory focus on implicit information preferences [*M*_happy_ = 0.11, *SD* = 0.38; *M*_sad_ = −0.08, *SD* = 0.37; *M*_prom_ = 0.06, *SD* = 0.41; *M*_prev_ = −0.02, *SD* = 0.36; *F*_(1,54)_ = 4.28, *p* < 0.05], and the main effect of emotions was also significant [*F*_(1,54)_ = 4.28, *p* < 0.05]. As in experiment 1, in order to further understand the effect of the interaction between emotions and regulatory focus on implicit information preferences, we conducted a simple effect analysis. The results indicated that, in the promotion-focused condition, there was a more significant difference for the influence of happiness on implicit information preferences in comparison to sadness [*M*_happy_ = 0.26, *SD* = 0.41; *M*_sad_ = −0.14, *SD* = 0.30; *F*_(1,54)_ = 8.21, *p* < 0.05]. That is, individuals preferred hedonic information when they are happy, but functional information when they are sad. However, in the prevention-focused condition, there was no significant difference for the influence of emotions on implicit information preferences [*M*_happy_ = −0.02, *SD* = 0.32; *M*_sad_ = −0.01, *SD* = 0.42; *F*_(1,54)_ = 0.00, *p* = 0.99].

**TABLE 3:** Analysis of variance of the effect of emotions and regulatory focus on information implicit preference.

Variables	*df*	MS	*F*	*p*	Partial *η*^2^
Emotion	1	0.56	4.21	0.05	0.07
Regulatory focus	1	0.08	0.58	0.45	0.01
Emotion × Regulatory focus	1	0.57	4.28	0.04	0.07
Error	54	0.13			

### Discussion

The results of experiment 2 indicated that, in the promotion-focused condition, the implicit effect that was triggered by happiness was significantly greater than the sadness-triggered effect. However, in the prevention-focused condition, implicit information preferences were not easily affected by emotions. We then focused on implicit effects to test hypothesis 1 again. The results suggested that emotions were good predictors of information preferences in the promotion-focused condition, but that they were not significantly related to information preferences in the prevention-focused condition.

The affect-as-information theory states that emotions can be used as “internal signals that provide consciously available feedback” (Clore et al., 2001, p. 30), and the effect of an emotion depends on how the experience of that emotion is attributed. When emotions are evoked by the target judgment, the internal signals provided by emotions are valid. However, when emotions are generated by other sources, people still associate them with the targeted task; thus, emotion-supplied internal signals can provide misleading information. Experiment 3 explored the effects of emotions and regulatory focus on information preferences when the emotion’s source was salient.

## Experiment 3

In experiment 3, we aimed to test hypothesis 2, whether the interaction between emotions and regulatory focus on information preference would be mitigated when the source of emotions was made salient.

### Participants and Design

The participants were 64 undergraduate and graduate students (32 women and 32 men), who were provided with small gifts worth CNY 15 yuan for their participation. During IAT, data from 57 participants were analysed, 7 were excluded due to unfinished implicit test questions. The participants were randomly assigned to a 2 (regulatory focus: promotion or prevention focus) × 2 (emotion: happy or sad) between-subjects design.

### Procedure

The experimental materials and procedures used in experiment 3 were similar to the ones used in experiment 2, with the following exceptions. The participants were asked to complete a questionnaire, adapted from Gasper and Clore (2000), designed to draw their attention to their emotions and to identify the possible source of their emotions. For example, one question is, “To what extent do you think you would feel differently now if you did not just recall the event or view the photo?” “After completing the questionnaire, the participants completed the information selection task and the IAT.

### Results

#### Information Selection Preferences

The results displayed in Table 4 indicated that when the emotion’s source is made salient, there is no significant interaction effect between emotions and regulatory focus on information selection preference [*M*_happy_ = 1.59, *SD* = 0.50; *M*_sad_ = 1.81, *SD* = 0.40; *M*_prom_ = 1.69, *SD* = 0.47; *M*_prev_ = 1.72, *SD* = 0.46; *F*_(1,60)_ = 1.92, *p* > 0.05]. The results suggested that the main effects of emotions and regulatory focus on information selection preferences were also insignificant.

**TABLE 4:** Analysis of variance of the effect of emotions (highlighting the source) and regulatory focus on information selection preference.

Variables	*df*	MS	*F*	*p*	Partial *η*^2^
Emotion	1	0.77	3.77	0.06	0.06
Regulatory focus	1	0.02	0.08	0.78	0.001
Emotion × Regulatory focus	1	0.39	1.92	0.17	0.03
Error	60	0.20			

#### Implicit Information Preferences

Table 5 displays the results for implicit information preferences obtained in experiment 3. The results indicated that when an emotion’s source is made salient, there is a significant interaction effect between emotions and regulatory focus on implicit information preferences [*M*_happy_ = −0.05, *SD* = 0.29; *M*_sad_ = −0.16, *SD* = 0.31; *M*_prom_ = −0.11, *SD* = 0.30; *M*_prev_ = −0.11, *SD* = 0.31; *F*_(1,53)_ = 9.19, *p* < 0.05]. The results also suggested that the main effects of emotions and regulatory focus on implicit information preferences were insignificant.

**TABLE 5:** Analysis of variance of the effect of emotions (highlighting the source) and regulatory focus on information implicit preference.

Variables	*df*	MS	*F*	*p*	Partial *η*^2^
Emotion	1	0.16	1.95	0.17	0.04
Regulatory focus	1	0.00	0.002	0.96	0.00
Emotion × Regulatory focus	1	0.73	9.19	0.004	0.15
Error	53	0.08			

### Discussion

The results obtained in experiment 3 showed that there was no interaction effect of regulatory focus and emotions on information preference when the source of the emotion was salient. These results are consistent with the affect-as-information explanation, and they verified hypothesis 2. In the salient emotion condition, emotions had less informational value for the current goal task (Scott and Cervone, 2002). However, our results indicated that, in the salient source condition, people’s emotions significantly interacted with the effect of regulatory focus on implicit information preferences. These results seem to contradict the emotional discounting effect. However, based on the affect-as-information theory, scholars have discussed that the discounting effect is based on the level of the participants’ consciousness rather than on their unconsciousness. This demonstrates that the mechanism of affect-as-information might be different for the level of implicit consciousness than it is for the level of explicit consciousness.

## General Discussion

### Summary of Findings and Implications

This research relied on the affect-as-information theory to examine the effects of regulatory focus and emotions on information preferences. The results obtained in experiment 1 indicated that promotion-focused participants were more likely to rely on emotions in selecting information in comparison to prevention-focused participants. The results specifically suggested that promotion-focused individuals preferred hedonic information when they are happy and functional information when they are sad. However, the results indicated that prevention-focused individuals’ emotions did not affect their information choices. Experiment 2 closely replicated experiment 1 and showed that emotions and regulatory focus affected implicit information preferences. As expected, people’s implicit information preferences were more strongly influenced by emotions in the promotion focused-condition than in the prevention-focused condition. These results are consistent with prior research. Pham and Avnet (2009) reported that promotion increases one’s reliance on emotions in making judgments, but that prevention decreased such reliance. When promotion-focused people are happy, they tend to focus on hedonic experiences that then take precedence over functional experiences (Wegener et al., 1995). However, when promotion-focused people are sad, they are more likely to be sensitive to future losses (Lench et al., 2011). In contrast, prevention-focused individuals with high risk aversions tend to decrease their reliance on emotions but increase their reliance on analytical processes (Friedman and Förster, 2000; Roy, 2017).

The objective of experiment 3 was to investigate the interactive effect of salient sources of emotions and regulatory focus on information preferences. The findings indicated that people’s emotions in the salient source condition did not interact with regulatory focus to influence information selection preferences. These results are consistent with previous findings and provide evidence for the affect-as-information explanation. However, it is noteworthy that salient sources of emotions and regulatory focus significantly interacted with and influenced implicit preferences. These findings seem to contradict the emotional discounting effect, which was discovered in people’s explicit consciousness. Our findings extend affect-as-information research to capture how salient source emotions are used in the realm of implicit consciousness. While previous studies claimed that emotions can provide information that affects subsequent judgments and decisions, we found that the effect of an emotion depends on how the emotion’s experience is attributed. That is, making the source of the emotion salient could mitigate its informational effect (Clore et al., 2001; Raghunathan et al., 2006; Isbell and Lair, 2013).

Experiment 3 demonstrated that salient emotions and regulatory focus did not generate any discounting effects at the implicit level. Thus, we might deduce that the affect-as-information model has different mechanisms in regard to an individual’s implicit and explicit mental activities. This experiment proved that explicit and implicit preference sometimes diverged. Explicit measures employed questionnaires or interviews, which may have shown only those attitudes that the individual considered to be favorable. Implicit measure tapped predominantly into automatic associations, which could capture unconscious attitudes (Steffens, 2004; Petty, 2006). But this does not deny the predictive validity of explicit measures. Explicit preferences better predict deliberate and controlled behaviors, and implicit preferences are valuable predictors of spontaneous or impulsive behaviors (Greenwald et al., 2009). In experiment 3, the IAT automatically measured implicit information preferences, and the results indicated that no discounting effect occurred in the salient condition. In any case, the findings in the present study suggest that future researchers should explore the mechanism of affect-as-information at the implicit level.

Our findings have important practical implications. We demonstrated that emotions interact with regulatory focus to influence information preferences. Enhancing one’s preference for certain information needs induces a corresponding emotion that matches with one’s regulatory focus. For promotion-focused individuals, using contextual cues to induce happiness (or sadness) can increase their preference for hedonic (or functional) information, assuming that the emotion’s source is not highlighted. Prevention-focused individuals’ information preferences are less affected by emotions. In sum, context-induced emotions ultimately influence the information preferences of promotion-focused, not prevention-focused, individuals.

### Limitations and Directions for Future Research

The research has several limitations. Firstly, in relying on the affect-as-information theory to verify that emotions interact with regulatory focus to affect information preferences, we did not identify either when the interaction occurs or what factors influence the effect. Future research should include more rigorous control conditions and more precise measurements to further investigate the interaction mechanism. Secondly, our laboratory experiments had participants complete tasks to prime their situational or temporary regulatory focus, but it is not clear whether trait or long-term regulatory orientation would have the same effect. In addition, it is also unclear whether temporary regulatory focus is affected by long-term regulatory focus, which is an area for subsequent studies to consider. Finally, this research used only student samples; while student samples provide suitable study groups for constructing or testing a theory, the homogeneity of such a sample limits generalizability. Future research should consider a more representative sample in order to improve the research’s external validity.

## Data Availability Statement

All datasets generated for this study are included in the article/supplementary material.

## Ethics Statement

Ethical review and approval was not required for the study on human participants in accordance with the local legislation and institutional requirements. Written informed consent to participate in this study was provided by the participants.

## Author Contributions

XW developed and designed the methodology, collected the data, and acquired the financial support for the project leading to this publication. QZ formulated the research goals and aims. YG prepared the published work and made statistical analyses. JW and JL conducted data collection and edited. All authors were involved in the writing of the theoretical background and discussion sections.

## Conflict of Interest

The authors declare that the research was conducted in the absence of any commercial or financial relationships that could be construed as a potential conflict of interest.
